# Testing Whether Humans Have an Accurate Model of Their Own Motor Uncertainty in a Speeded Reaching Task

**DOI:** 10.1371/journal.pcbi.1003080

**Published:** 2013-05-23

**Authors:** Hang Zhang, Nathaniel D. Daw, Laurence T. Maloney

**Affiliations:** 1 Department of Psychology, New York University, New York, New York, United States of America; 2 Center for Neural Science, New York University, New York, New York, United States of America; University College London, United Kingdom

## Abstract

In many motor tasks, optimal performance presupposes that human movement planning is based on an accurate internal model of the subject's own motor error. We developed a motor choice task that allowed us to test whether the internal model implicit in a subject's choices differed from the actual in isotropy (elongation) and variance. Subjects were first trained to hit a circular target on a touch screen within a time limit. After training, subjects were repeatedly shown pairs of targets differing in size and shape and asked to choose the target that was easier to hit. On each trial they simply chose a target – they did not attempt to hit the chosen target. For each subject, we tested whether the internal model implicit in her target choices was consistent with her true error distribution in isotropy and variance. For all subjects, movement end points were anisotropic, distributed as vertically elongated bivariate Gaussians. However, in choosing targets, almost all subjects effectively assumed an isotropic distribution rather than their actual anisotropic distribution. Roughly half of the subjects chose as though they correctly estimated their own variance and the other half effectively assumed a variance that was more than four times larger than the actual, essentially basing their choices merely on the areas of the targets. The task and analyses we developed allowed us to characterize the internal model of motor error implicit in how humans plan reaching movements. In this task, human movement planning – even after extensive training – is based on an internal model of human motor error that includes substantial and qualitative inaccuracies.

## Author Summary

When you play darts, which part of the dartboard do you aim at? The tiny bull's eye is worth 50 points. The 20-point section is much larger. If you're not very good at dart throwing you may want to take that into account. Your choice of target depends not on how good you are but on how good you *think* you are – your internal model of your own motor error distribution. If you think you hit exactly where you aim, you should aim at the bull's eye. If you assume you have less error in the horizontal direction, you would tend to choose vertically elongated targets over horizontally elongated ones. Previous work in movement planning hints that people have accurate models of their own motor error; we test this hypothesis. People first practiced speeded reaching to touch targets. We then asked them to choose between targets of varying shapes and sizes. Their pattern of choices allows us to estimate their internal models of their own motor error and compare them to their actual motor error distributions. We found – in contrast to previous work – that people's models of their own motor ability were markedly inaccurate.

## Introduction

Human movement is prone to error. This error may be reduced after extensive practice or under careful control, but can never be entirely eliminated. It can have severe consequences when, for example, the outcome of a surgical procedure hangs on the accuracy of the surgeon's movements. Human decisions often reflect an internal model of the probabilistic regularities of the world [1]. We would expect to find such an internal model of the uncertainties in our own movements.

Indeed, the unpredictable error inherent in movement has provided a rich and precise laboratory model of decision under uncertainty. Recent studies have shown that human decisions under visual and motor uncertainty are close to those predicted by Bayesian Decision Theory, maximizing expected gain [2]–[9]. However, these studies are not particularly sensitive tests of subjects' knowledge of their own distributions.

**Figure 1:**
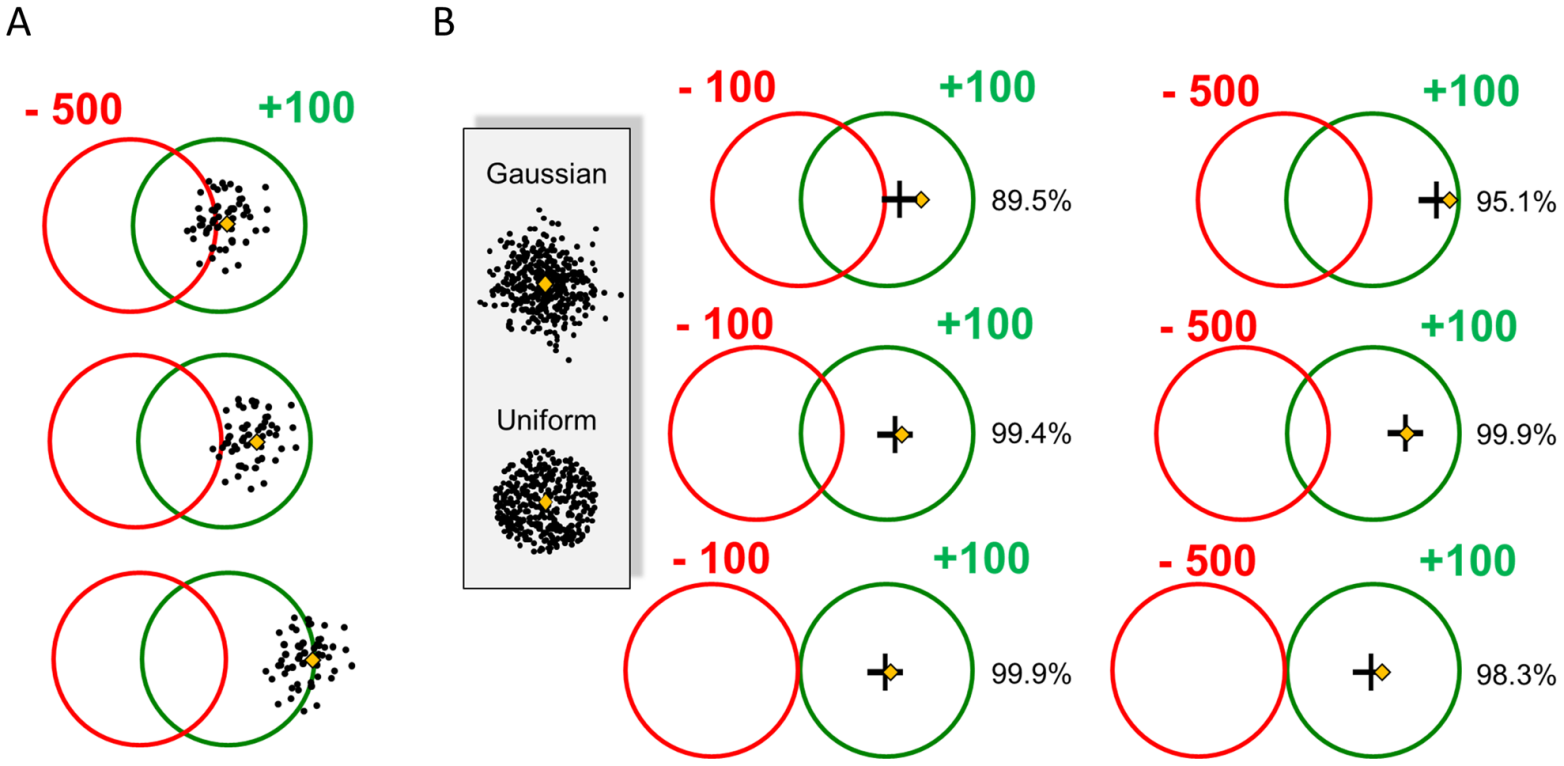
Performance of a hypothetical subject in Trommershäuser et al.'s [7] experiment. The subject made speeded hand movements to the target (green circle) for rewards. The red circle denotes the penalty region. The experiment had six conditions: the penalties were either −100 or −500, and the distance between the two circles is one of 1, 1.5, or 2 times of the radius of the circle. The radii of all circles were 8.97 mm. The reward for hitting the green circle was always +100. Falling outside both circles led to 0 reward. A. Consequences of possible aim points. The subjects' actual error distributions were indistinguishable from isotropic, bivariate Gaussian distributions. The aim point is shown in gold in three examples. A possible distribution of end points of actual reaches is shown around each end point (SD 4.05 mm). Each end point incurs a penalty or reward (or both) depending on where it falls within the red or green regions. In the topmost example, the subject is likely to incur many large penalties. In the bottommost, the subject incurs few penalties but on many trials the end point falls outside both circles and incurs 0 reward. The golden diamond in the middle diagram is the aim point that maximizes expected gain for an subject with this error distribution and the rewards and stimuli shown. B. A hypothetical observer with an erroneous model. The hypothetical subject correctly estimates her SD 4.05 mm but makes an erroneous assumption about the shape of her error distribution around the aim point. She assumes that it is a circular, uniform distribution rather than a bivariate Gaussian error distribution and plans her movements accordingly. The golden diamond denotes the aim point based on the true Gaussian that would maximize expected gain in each condition. The black cross denotes the hypothetical subject's choice of aim point based on her erroneous distributional model. The percentage on the right of each panel denotes the expected gain of the hypothetical subject divided by the maximum expected gain possible. Note that the hypothetical subject's performance is not far from optimal.

In one early study, for example, Trommershäuser, Maloney, & Landy [7] asked human subjects to make speeded reaching movements to a touch screen. There were two partly overlapped circular regions on the screen (Figure 1A). A touch within the green region earned a reward, within the red, a penalty. Any end points outside of both regions earned neither reward nor penalty. The challenge to the subject was to decide where he should aim in order to maximize his expected winnings.

In Figure 1A we illustrate three possible aim points (golden diamonds) and a realization of movement end points around the aim point. The aim point in the upper configuration is so close to the red penalty circle that the penalty is incurred on a high proportion of trials. In contrast, the aim point in the lower configuration is far from the penalty area and it is unlikely that the subject will incur a penalty on any given trial. However, on many trials, her end point falls outside of both circles and she earns no reward for her effort.

The aim point that maximizes expected gain for the subject with this motor error distribution is shown in the middle configuration: it is away from the center of the rewarding region in the direction opposite to the penalty region. Its position depends on the subject's error distribution, the locations of reward and penalty regions, and the magnitudes of rewards and penalties. Trommershäuser et al. [7] found that human subjects shifted their aim points with varying reward conditions and the amount of rewards they won were close to that predicted by an optimal choice of aim point, ranging from 92.0% to 106.9% of the latter for different subjects. The implication is that people can compensate for their motor uncertainty in order to maximize monetary gain. Given this result and similar results found in the literature, it is tempting to assume that human movement planning is based on an accurate model of motor uncertainty.

One goal of the present study is to interrogate and ultimately challenge this assumption. One reason to do so is that previous evaluations of human performance are not sensitive to even gross errors in the representation of motor uncertainty. In Trommershäuser et al.'s [7] experiment, for example, human subjects' end points on the screen formed a bivariate Gaussian distribution centered at the aim point. Suppose a subject correctly estimates the variance of the end points but mistakenly assumes that the end points are distributed uniformly in a fixed circle around the aim point. This subject has a model of his own error distribution that is markedly different from her actual error distribution. The two distributions are illustrated in an inset to Figure 1B.

To evaluate the performance of such a hypothetical subject, we simulated the six reward conditions of Trommershäuser et al. [7] and plotted the results in Figure 1B. The differences between the optimal aim point (golden diamond) and the aim point of the hypothetical subject (black cross) is small, less than 1 mm on average and the average expected gain of the hypothetical subject was as high as 97.0% of the maximum expected gain. That is, although the hypothetical subject had an inaccurate model of her own error distribution, her performance would probably be indistinguishable from optimal in Trommershäuser et al.'s [7] experiment, and in any of the studies we cited earlier.

We developed a simple motor choice task to more directly assess humans' internal models of their own motor error distributions. Human subjects were first trained to make speeded movements to radially-symmetric targets on a computer display. They were permitted only a short time to execute the movement and hit the screen. During the training, we estimated subjects' true motor error distributions 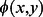. They were all well described as vertically elongated, bivariate Gaussian distributions.

**Figure 2:**
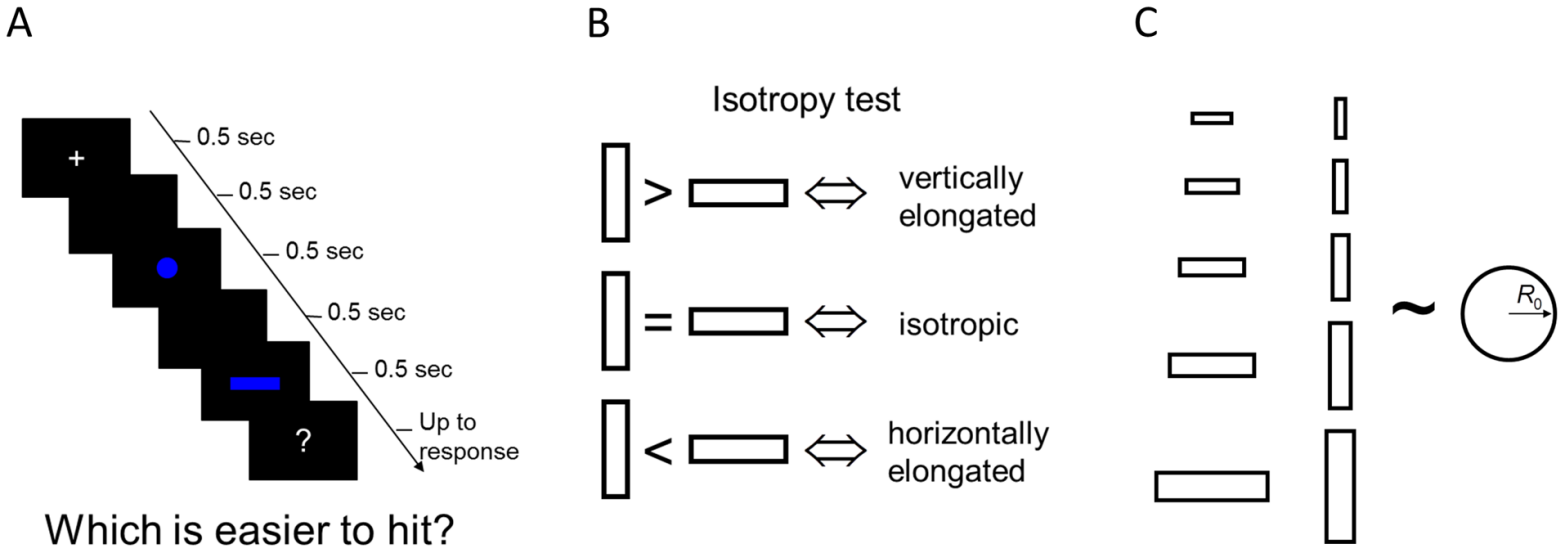
The choice task. A. Example of a trial. A rectangle and a circle were displayed sequentially. Subjects were prompted “which is easier to hit? 1^st^ or 2^nd^?”. B. Illustration of the isotropy test. One way to test the isotropy of subjects' model of the motor error distribution is to compare subjects' equivalent radius for horizontal and vertical rectangles of the same size. It amounts to comparing the “hittability” of horizontal and vertical rectangles. If the subject correctly assumes that the end points of her movement are vertically elongated, the subject would judge the vertical rectangle as easier to hit than the horizontal one. Instead, the assumption of an isotropic distribution would lead to indifference between the two; the assumption of a horizontally elongated distribution, a preference favoring the horizontal rectangle. C. Conditions. In Experiment 1, the rectangle in a pair had two possible orientations and five possible sizes. For each rectangle the radius of the paired circle was adjusted by an adaptive staircase procedure to obtain the equivalent radius, 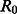. The set of rectangles used in Experiment 2 was similar except that there were four instead of five possible sizes. See Methods.

In the second phase of the experiment, subjects did not attempt to hit targets. Instead they were given pairs of potential targets, one rectangle and one circle, of specific sizes. The task was to choose the target that was easier to hit (Figure 2A). Subjects knew that at the end of the experiment they would attempt to hit a small number of the targets they had chosen and they would be paid a cash reward for each success. The cash reward for either target was the same and it was therefore in their interest to choose the target in each pair that offered the higher probability of success.

If the targets are denoted 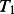 and 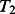 then the true probability of success in hitting the *i^th^* target is
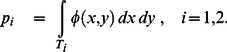
(1)The target is just the region of integration and the probability of success is just the proportion of the probability density function contained within the target. (We verified in training that subjects aimed at the centroid of the targets.)

But how is the *subject* to decide between targets? We consider the possibility that she has some internal estimate of the distribution of her own motor uncertainty, 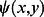. In evaluating each target, she computes an estimate of probability based on this estimate,
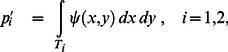
(2)and then chooses whichever target offers the higher probability.

This strategy would maximize expected gain in our task if the subject's estimate of her distribution were accurate: 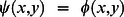. If it is not, then some of her choices will differ from the choice dictated by Eq. 1.

We illustrate the method by explaining how we test for isotropy (Figure 2B). Suppose the subject is given two rectangles, one horizontal and one vertical, of the same size and is asked to choose the one that is easier to hit. If the subject's internal model is an isotropic distribution, i.e. equal variance in the horizontal and vertical directions, the subject should be indifferent between the two rectangles. Alternatively, if the subject assumes a horizontally elongated distribution, i.e. a larger variance in the horizontal distribution, the subject would prefer the horizontal rectangle, and vice versa. (In practice, we never asked subjects to directly compare a horizontal rectangle and a vertical rectangle. Instead, we used a staircase method to determine the radii 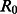 of the circle that the subject judged to be as “hittable” as any given rectangle and compared these equivalent radii.)

Ten (Experiment 1) or eight (Experiment 2) different rectangles, horizontal or vertical, were used and for each we measured its equivalent radius 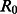, where the subject chose indifferently between the rectangle and the circle (Figure 2C). Based on a subject's choices for varying pairs of targets, we are able to test the variance and anisotropy of her distribution model.

In Trommershäuser et al.'s [7] task, subjects were facing “motor lotteries” with the probabilities of different outcomes determined by their own motor error. One concern is the possible effect of probability distortions on the interpretation of these studies. It is well-known that humans overweight small probability and underweight large probability in classical decision tasks [10], where they choose among economic lotteries. Wu, Delgado, and Maloney [11] show that people have systematic probability distortions with motor lotteries as well, although in a reverse pattern: they underestimated small probabilities and overestimated large probabilities. Choice between targets in our task depends only on ordering of the estimates of the probabilities of hitting them – since the reward associated with success never varies – and is thus insensitive to any distortion of probability. In particular, if 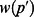 is any strictly increasing function of probability 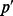 which the subject applies to the probabilities computed from Eq. 2, then 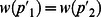 precisely when 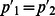.

## Results

We report the results of two experiments. For simplicity, we focus on Experiment 1 and use Experiment 2 to address concerns raised in Experiment 1. We first describe the true motor error distributions measured in the training task. We then fit subjects' responses in the probability choice task to a probabilistic model with two free parameters and compared subjects' models to those of their true motor error distribution. We report large, systematic deviations. Particular patterns in subjects' model failures are identified. Unless otherwise stated, the significance level used was .05.

### Experiment 1

#### True motor error distribution

The true error distribution depends on the trajectory of the movement, which we controlled by ensuring that participants started all reaches from a common starting point (the space bar of our computer keyboard). Additionally, the subject has some control over her motor error distribution 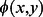. Normally, she can move more or less quickly, altering 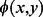, and previous research demonstrates that humans do trade speed for accuracy, information or reward [2]–[4]. By imposing a time limit on the movement (from release of the space bar to touch of the screen) we effectively eliminated this freedom.

**Figure 3:**
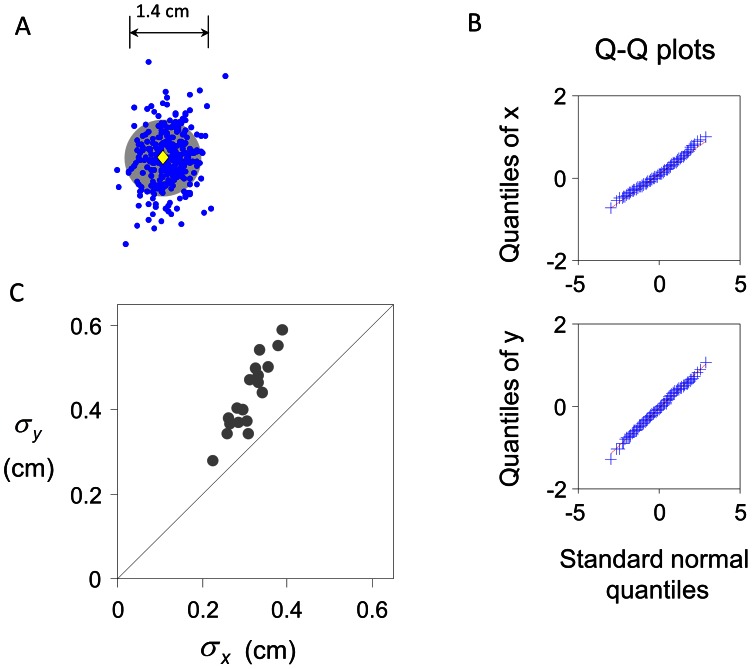
True error distribution in Experiment 1. A. End points of one subject in the training task. Blue dots denote end points. The grey filled circle denotes the target. Yellow diamond marks the centroid of the target. The distribution of the errors is bivariate Gaussian, elongated in the vertical direction. B. Q-Q plots for the end points of the subject. The quantiles of the horizontal and vertical positions of the end points are plotted against the standard normal quantiles. The linear relationship in both directions implies that the distribution of the end points is bivariate Gaussian. C. The standard deviations of the end points in the horizontal (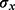) and vertical (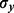) directions. Each point is for one subject. All subjects had a larger 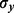 than 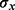. That is, their distributions were vertically elongated. The median 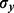-to-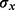 ratio across subjects was 1.44.

Only movements completed within the time limit were included into analysis. The end points for a typical subject are shown in Figure 3A.

We first examined Q-Q plots of all subjects' end points; as in past work, the Q-Q plots were close to linear, indicating that subject's motor errors were close to bivariate Gaussian. In Figure 3B we show the Q-Q plots for one typical subject.

For each subject, we fitted the 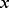 and 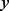 coordinates of the endpoints to a bivariate Gaussian distribution. For simplicity, we treated the 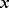 and  errors as independent (uncorrelated) and estimated 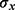 and 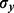 by computing the standard deviation separately for the horizontal and vertical direction. However, for 10 of the 18 subjects, the errors in the 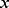 and 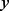 directions were significantly correlated (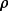 ranged from −0.44 to 0.33). We verified that the slight “tilt” introduced by correlation (e.g. Figure 3A) had negligible effect on any of our further tests: Taking into account the correlation would have no influence on the probability of hitting circles and would change the probability of hitting any of the rectangles we tested by no more than 3%.

Figure 3C shows the relationship between 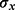 and 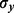. We tested for equality of variance separately for each subject (one-tailed *F* test). All subjects had a significantly larger 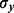 than 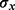: the distribution was vertically elongated. The median 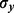-to-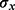 ratio across subjects was 1.44.

In summary, subjects' estimated motor error distributions were vertically elongated bivariate Gaussians. If we define the *variance parameter* 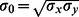 and the *anisotropy parameter* 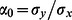, the motor error distribution can be written as:
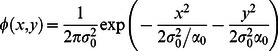
(3)


#### Subjects' model: Gaussian vs. area-matching

**Figure 4:**
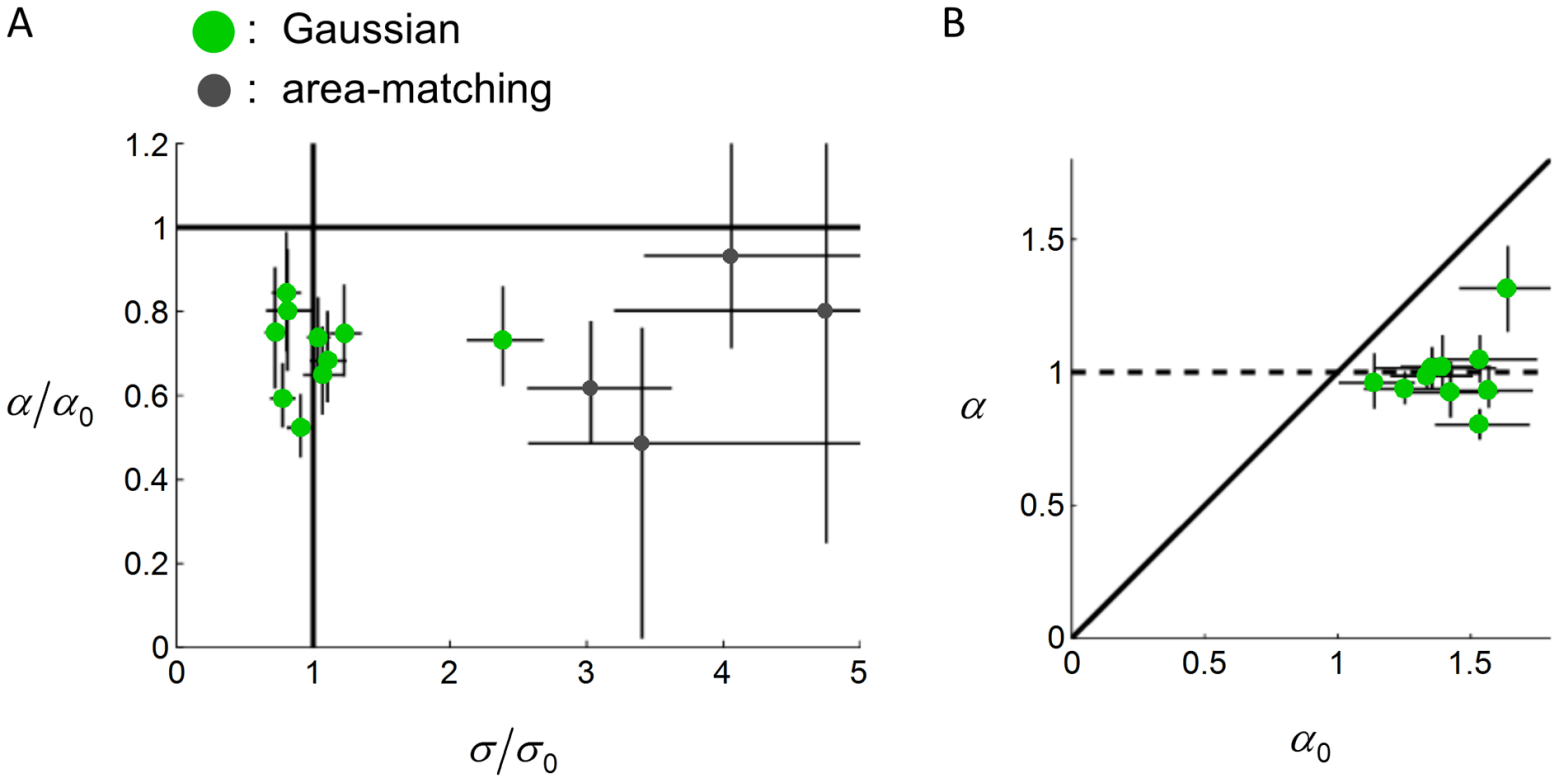
Subjects' models in probability choices in Experiment 1. Each circle denotes one subject. Error bars denote 95% confidence intervals. 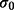 and 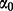 are the variance and anisotropy parameters of the true distribution 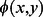 (Eq. 3). 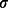 and 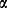 are their counterparts in the subject's model 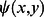 (Eq. 4). A. 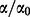 plotted against 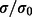 **.** Among the 18 subjects, 10 subjects (the Gaussian type, in green) were better fit by the Gaussian model, who had internal variance close to true (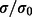 close to 1) but who underestimated the vertical anisotropy of their true distribution (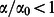). The remaining 8 subjects (the area-matching type, in gray) were better fit by the area-matching model, as if they were comparing the areas rather than the probabilities of hit of the targets. Four subjects of the area-matching type resulted in too large 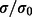 (13, 19, 57, 889) and were not plotted. b. 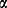 plotted against 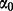 for subjects of the Gaussian type. Note that 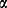 was close to 1 regardless of the value of 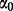 for most subjects. That is, the distribution was incorrectly assumed to be isotropic in subjects' model.

To allow for concrete parametric comparisons, we estimate the subjects' models of their own motor error distributions, 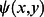, assuming they have the same Gaussian form as 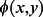 but with possibly different variance and anisotropy parameters:
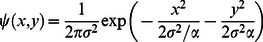
(4)(We consider the possibility of other distributional forms in the discussion.) Based on their choices, we estimated each subject's 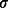 and 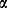 (see Methods). Figure 4A shows the estimates of 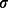 and 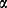, relative to 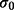 and 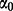.

We considered the possibility that some subjects were choosing the target of larger area rather than the target of larger probability of a hit. If a subject used this area-matching strategy, his estimated 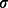 would approach infinity. Indeed, the estimated 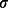 of a considerable number of subjects were far greater than 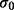, up to 889 times of 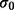.

For each subject, we tested the Gaussian model (Eq. 4) against an area-matching model (see Methods). The area-matching model could be treated as a special case of the Gaussian model with 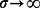 and 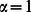. According to nested hypothesis tests [12], 10 out of 18 subjects were better fit by the Gaussian model and the remaining 8 subjects were better fit by the area-matching model. For convenience, we call the former the Gaussian type, the latter the area-matching type.

#### Subjects' model: variance and anisotropy

We explored the variance and anisotropy of the subjects of the Gaussian type (Figure 4A, in green; we did not further examine the parameter estimates for subjects of the area-matching type). If a subject's model were the same as her true motor error distribution, her data point in Figure 4A would fall on the coordinate (1, 1). According to the 95% confidence intervals of 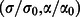, all the subjects' models deviated from their true motor error distributions.

For a bivariate Gaussian distribution, the central regions have higher probability density than peripheral regions. Because circles are more concentrated than rectangles, a circle that is as equally “hittable” as a specific rectangle should be smaller than the rectangle in area. Intuitively, the larger the variance parameter 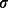, the more dispersed the assumed distribution, the larger the equivalent radius for a specific rectangle.

For most of the subjects of the Gaussian type, the estimated internal variance was close to their true variance (Figure 4A). For 4 out of these 10 subjects, 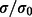 was not significantly different from one. Four subjects' 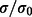 were significantly less than one and two subjects, significantly greater than one.

The anisotropy parameter 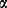 determines the perceived relative “hittability” of horizontal and vertical rectangles, as we illustrated in the Introduction. If two rectangles have the same size, the larger the 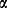, the larger the equivalent radius of the vertical rectangle relative to that of the horizontal rectangle.

All the subjects of the Gaussian type underestimated the vertical anisotropy of their true distribution, with all their 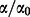 significantly less than one. We plotted 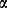 against 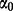 (Figure 4B) and further examined whether subjects' 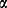 was sensitive to their true anisotropy 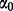. Subjects' 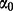 varied from 1.14 to 1.64. There was no significant correlation between 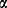 and 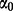, Pearson's *r* = .34, *p* = .34. Instead, 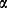 was always close to one. For 8 out of the 10 subjects, the 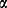 was indistinguishable from one. That is, most of the subjects of the Gaussian type incorrectly treated their error distribution as isotropic.

To summarize, there were two patterned biases in subjects' models in the probability choice task: First, approximately half of the subjects failed to take their own motor error distributions into account and evidently based their choices on the areas of the targets instead. Second, among the subjects who correctly assumed a Gaussian model, 4/5 of them incorrectly assumed the vertically-elongated distribution to be isotropic.

#### Results of the area choice task

As a control for the probability choice task, in a subsequent area choice task, subjects were asked to choose which target was larger in area. We investigated whether the Gaussian and area-matching subjects also differed in their judgment of area.

**Figure 5:**
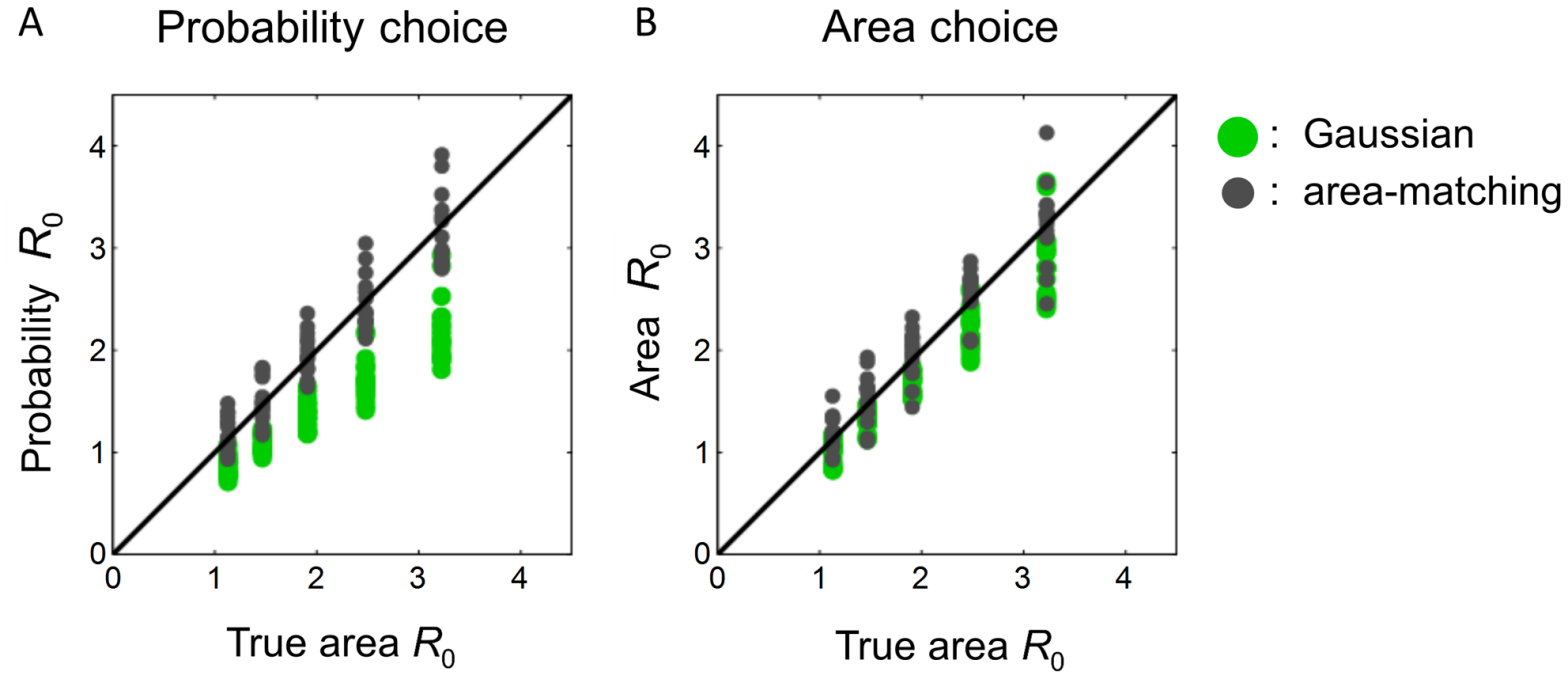
Probability vs. area choices in Experiment 1. Equivalent radius in the probability choice task (A) and in the area choice task (B) are plotted against the true area equivalent radius. Each dot is for one subject and one rectangle condition. Green denotes subjects of the Gaussian type. Gray denotes the area-matching type. Note that the probability choices of the area-matching type improperly agreed with the predictions of area-matching, while the area choices of the Gaussian type improperly deviated from the true area equivalent radii.

In the area choice task, the equivalent radii of subjects of the area-matching type were close to the true area equivalent radii (i.e. area-matching) while subjects of the Gaussian type had smaller equivalent radii than the true (Figure 5B). This separation resembled that in the probability choice task (Figure 5A).

For probability choice, the reason equivalent radii are expected to be smaller than true radii is that the probability of hitting a circle is larger than hitting a rectangle equal in area: a circle fits more compactly near the center of an isotropic Gaussian error distribution. This effect reflects the implicit motor model because the radius difference is most dramatic for sharper Gaussians (smaller 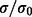) and vanishes in the limit of the infinite variance Gaussian (i.e., area matching). Accordingly, that their equivalent radii are smaller even in the area choice task suggest that subjects of the Gaussian type compensated for the probabilities of hitting targets even when judging area, although not as much as they did in the real probability choice task: their median 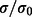 was 0.97 in the probability choice task and 2.29 in the area choice task.

Indeed, we found that 8 out of 10 Gaussian subjects' area choices were better fitted by the Gaussian model than by the (now appropriate) area-matching model. In contrast, only 2 out of 8 area-matching subjects were better accounted by the Gaussian model. Subjects of the Gaussian type had a significantly larger proportion to incorrectly assume Gaussian in the area choice task, according to a Fisher's exact test.

We verified that subjects were not just confusing the probability and area choices and they did have different equivalent radii (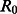) in the two tasks. For each subject, we submitted the log differences between their probability 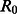
's and area 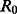
's to a one-sample two-tailed Student's *t*-test. The probability 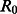
's were indistinguishable from the area 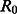
's for only one subject who was of the area-matching type. The probability 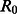
's were significantly smaller than the area 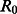
's for all the 10 subjects of the Gaussian type and 4 subjects of the Gaussian type. The remaining 3 subjects of the area-matching type had the probability 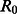
's significantly larger.

### Experiment 2

In Experiment 1, subjects' models of their own motor error distributions had patterned deviations from the true distributions. In Experiment 2, we tested whether these deviations could be eliminated or reduced by two manipulations.

First, we tested the effect of having more reaching experience with a single target, which might be expected to improve subjects' motor models. In the training phase of Experiment 1, subjects made 300 speeded reaches to a circular target of fixed size. We doubled the training trials to 600 in Experiment 2. As in Experiment 1, all subjects' true distributions were vertically elongated, bivariate Gaussian distributions.

Even with this more extensive training, among 12 new subjects, there were 2 subjects who were better fit by the area-matching model. Although this proportion is numerically smaller than that in the first experiment, the difference was statistically insignificant, according to a Fisher's exact test.

**Figure 6:**
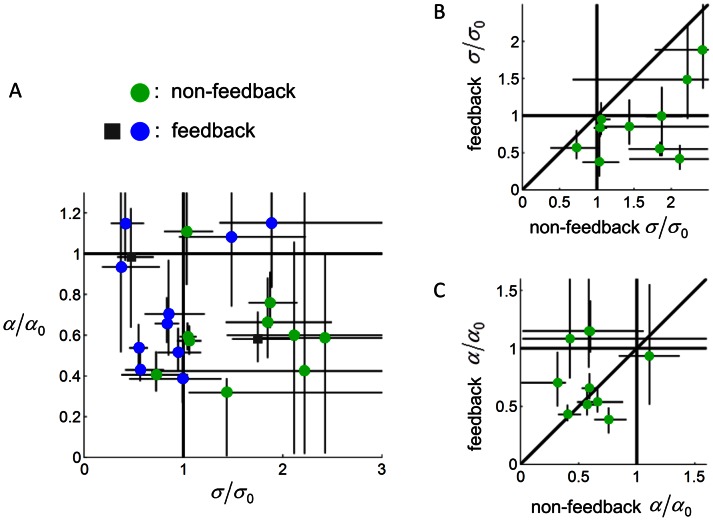
Subjects' model in Experiment 2. A. 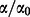 plotted against 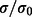 **.** Each circle or square denotes one subject. Green circles denote the non-feedback task (the two area-matching subjects, whose 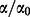 were 15 and 1210, were not plotted). Blue circles and gray squares respectively denote the performances in the feedback task for subjects who belonged to the Gaussian type and the area-matching type in the preceding non-feedback task. B. 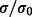 in the feedback task against 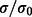 in the non-feedback task. C. 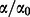 in the feedback task against 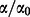 in the non-feedback task. Error bars denote 95% confidence intervals.

For the 10 subjects of the Gaussian type (green circles in Figure 6A), 5 subjects' 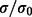 were indistinguishable from one and 5 subjects' 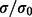 were significantly greater than one.

As in Experiment 1, most of these subjects, though not all, underestimated the vertical anisotropy of their distributions. Six out of the 10 close-to-one subjects' 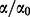 were significantly less than one (Figure 6A). Four subjects' 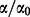 were indistinguishable from one, but among them, three had 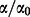 between 0.42 and 0.60 – the inability to reject the null hypothesis was probably just because of the inaccuracy of the measurement (see Figure S1 for an illustration).

The second manipulation we added in Experiment 2 was to include a final experimental phase in which subjects would choose which of two targets they would prefer to hit and, immediately after choosing the target, subjects would actually make a speeded reach to the target and watched its consequence. This task gave subjects an opportunity to observe the probability of hit of targets of different shapes and sizes. It came after the probability choice phase (which was conducted, as before, with no feedback), so it would have no influence on the results of the latter that we reported above.

In the feedback phase, none of the 12 subjects were better fit by the area-matching model. All the subjects' 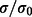 were less than those in the non-feedback task (Figure 6AB). Six of the 12 subjects now significantly underestimated their variance.

As to 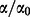 (Figure 6C), 4 out of the 6 subjects who significantly underestimated the vertical anisotropy in the non-feedback task had no improvement (those whose error bars cross the identity line) and one subject performed even worse (those whose error bars are under the identity line).

To summarize, receiving feedback on a variety of targets appears to correct the area-matching strategy but does not improve underestimation of variance or anisotropy.

## Discussion

We reported two experiments testing whether humans have accurate internal models of their own motor error distributions. In the first part of the experiments, subjects executed the reaching movements hundreds of times, allowing us to measure the error distribution of their end points. Though differing in variance, the distributions were all well characterized as bivariate Gaussian, all elongated in the vertical direction. Subjects were moving from a keyboard below the screen to the screen. A larger variance along the direction of movement (upward) is often reported [13]–[15].

Next, subjects were asked to repeatedly choose between two targets, selecting the one that appeared easier to hit. Based on their choices we tested two aspects of their internal model of their own distribution, variance and anisotropy.

The two experiments led to converging results: More than half of the subjects had accurate or almost accurate estimation of their own variance (no more than twice and no less than half of the true variance), while the rest failed badly, markedly overestimating their own variances or not taking into account their variance at all. Almost all the subjects failed to have an accurate estimate of how their distribution was shaped, incorrectly assuming a more isotropic distribution.

These failures are unexpected. Previous studies have shown close-to-optimal compensation for motor uncertainty [2]–[8]. In most of the studies [e.g. 7], subjects' decision under motor uncertainty was indicated implicitly by their movement. But close-to-optimal compensation was also observed in tasks resembling ours where explicit choices between two alternative options were required [16]. Patterned failure in compensating for motor uncertainty has seldom been reported.

As an exception, Hudson, Tassinari, & Landy [17] added anisotropic noise to the visual feedback of subjects' movements and found that people ignored the induced anisotropy. Our results in testing isotropy are consistent with theirs. But in their case, the artificial visual feedback conflicted with subjects' sensorimotor feedback. It is possible that subjects were just giving a higher trust to their own sensorimotor feedback. Our results are free of such possibilities: people do not correctly compensate for anisotropy even when the anisotropy emerges naturally.

The finding that a considerable proportion of subjects did not base their choices of movements on a model of their own distribution is unexpected and striking. However, it is not necessarily in conflict with previous studies where human performance is found to be close to optimal [7]. In previous studies, subjects performed real movements and received feedback in the test task as in the feedback task of our Experiment 2, where no subjects followed the area matching strategy.

In specific task situations, people can compensate for a missing or inaccurate model of their motor error distribution by using intuitive strategies. This is demonstrated in an anomaly reported by Wu et al. [18]. They used the same task as Trommershäuser et al. [7] with a different reward landscape and found sub-optimal human performances. In Trommershäuser et al. [7], the optimal aim point fell on the symmetrical axis and within the rewarding circle, both of which were highly intuitive. In contrast, Wu et al. [18] used an asymmetrical reward landscape that consisted of one rewarding circle and two penalty circles. The optimal aim point fell within one of the penalty circles. Subjects' failure in this counter-intuitive situation suggests that apparently optimal performances may rely on simple intuitive strategies.

An underestimation of variance, observed for half of the subjects even with feedback (Experiment 2), is probably not as costly to the same extent as an overestimation in the sorts of tasks considered by Trommershäuser and colleagues. Subjects in Trommershäuser et al.'s [7] task also received feedback after every trial and potentially this led to underestimation of variance in that experiment. We considered whether a considerable underestimation of their own motor variance could be compatible with humans' close-to-optimal performances. As we stated in the Results, in Trommershäuser et al.'s [7] task, if subjects overestimated their variance to up to 4 times the true variance (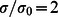), their expected gain would be only 74% of the maximum expected gain. To our surprise, however, if subjects underestimated their variance to 1/4 of the true variance, (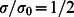), their expected gain would be as high as 96% of the maximum expected gain. That is, at least in this situation, an underestimation of variance incurred little penalty.

Subjects' failures in our experiment could not be attributed to a mere lack of experience. Before the two choice tasks, subjects repeated the reaching movements for over 300 or 600 times and the position of the endpoint was provided after each reach. Moreover, goal-directed reaching is arguably among the most practiced motor tasks of everyday life. Therefore, it is a mystery that people are not able to model their own motor error correctly and exhibit considerable and patterned deviations. Whence come their incorrect models? When will incorrect models be abandoned and be replaced by the correct ones?

For example, why did subjects assume an isotropic distribution? We conjecture that they were trying to use a model that is as simple as possible to fit their observations. When observations are few even a real isotropic distribution may be better fit by an anisotropic model. Thus, by adopting a simpler model, subjects could avoid over-fitting their observations. The problem is: why should people stick to an incorrect model even after hundreds of observations?

Another intriguing fact is the double dissociation between subjects of the Gaussian type and subjects of the area-matching type. For the area-matching subjects, area-matching seems to be their substitute strategy for the probability choice task; while for the Gaussian subjects, “probability-matching” seems to be their substitute strategy for the area choice task. If we consider the area of a target to be the integration of a unit probability density across the target region, area choices are comparable to probability choices. Is there any common process involved?

The choice task we designed in the present study is a powerful tool for determining what people “know” about their motor error (or more precisely, what model of motor error is consistent with their choice performance). It only asks for an ordering of probabilities. It does not depend on a utility function, since the two targets they are choosing from are associated with the same amount of reward. It is not influenced by how probability is non-linearly distorted [10], [19], so long as the distortion function retains the order of the probability scale.

Our task differs from most other motor decision tasks [e.g. 2], [7], [9] in two respects that might in principle produce different results: the choices are binary rather than continuous, and concern hypothetical future rather than immediately actualized movements. For example, people may not have full access to their motor uncertainty in the absence of real movement planning or execution. The existing evidence, however, does not suggest human choices in a binary, hypothetical motor task would necessarily differ from those in continuous, actualized movements. First, close-to-optimal performances were found in previous studies on binary, hypothetical motor decisions [16]. Second, the neural circuits activated by real and imagined movements are highly similar [20], [21].

In the present study, we estimated subjects' behavior using a Gaussian distributional assumption to allow direct quantitative comparison with the ideal observer model in terms of that model's parameters. Of course, it is possible that subjects assumed a different distributional form subjectively, or even chose based on some heuristic that does not directly correspond to the decision theoretic model for any distribution. Although both of these possibilities are interesting hypotheses for the source of the sub-optimality we reveal, even if true they would not invalidate the results of the present analysis in using the Gaussian fits descriptively to characterize the existence and nature of deviation from the ideal observer. As pointed out by Geisler [22], it is valuable to compare actual to ideal even when people are not ideal.

What distinguishes our study from previous studies is an exploration of the most likely model implicit in each individual's performances. We broke down the ideal observer into multiple dimensions (variance and anisotropy) and assessed human observers on these dimensions. The multi-dimensional tests accommodate the possibility that a specific individual may deviate from the ideal observer on some dimensions but not others, which a one-dimensional test would not afford. The deviation on each dimension is separable in subjects' choices. Our task is thus sensitive to the each particular individual's possible deviations from ideal and provides alternative models to ideal.

In a recent article [23] we found people do not have an accurate model of their own visual uncertainty. Subjects chose between visual discrimination tasks that could differ in location (retinal eccentricity) and contrast. By examining subjects' choices we could test what they implicitly assumed about their own retinal sensitivity in the periphery. We found that all but one subject was not even consistent in their choices: the pattern of choices violated transitivity of preference, i.e. in some cases they preferred lottery A over lottery B and lottery B over lottery C but, finally, lottery C over lottery A.

Had we simply compared subjects' performances to optimal in Zhang et al [23] and the present paper, we would only have concluded that subjects' performance was less than ideal, overlooking the striking patterns of failure and individual differences that we instead found.

## Methods

### Ethics statement

The experiment had been approved by the University Committee on Activities Involving Human Subjects (UCAIHS) of New York University and informed consent was given by the observer prior to the experiment.

### Experiment 1

#### Subjects

Eighteen subjects, ten female and eight male, participated. All had normal or corrected-to-normal vision and were not aware of the purpose of the experiment. Fourteen of them were right-handed and four left-handed. All used the index finger of the dominant hand for the speeded reaching task. Subjects received US$12 per hour plus a performance-related bonus.

#### Apparatus and stimuli

Stimuli were presented in a dimly lit room on a 17″ (33.8×27 cm) Elo touch screen mounted vertically on a Unistrut frame. The display was controlled by a Dell Pentium D Optiplex 745 computer using the Psychophysics Toolbox [24], [25]. The end points of subjects' reaching movements to targets on the touch screen were recorded by the touch screen. To optimize the recording accuracy, a touch screen calibration procedure was performed for each subject.

Subjects were seated at a viewing distance of 30 cm with the aid of a chinrest. Blue filled shapes appeared on a black background. The shape in all tasks was positioned at the center of the screen offset by a small random jitter uniformly distributed in the range of ±1 cm horizontally and vertically. Subjects started their reach from a key on the keyboard, which was 28 cm away from the screen in depth and 20.5 cm below the screen center. The total distance from starting point to the center of the target was 34.7 cm.

#### Procedure and design

Subjects completed three tasks in a sequence: *training*, *probability choice*, and *area choice*. The training task took approximately 25 min. Each choice task took approximately 80 min. The training and probability choice tasks were conducted in the first session. The area choice task was conducted in a second session on a different day.

Training. The training task was to touch a circular target (1.4 cm in diameter) on the touch screen within 400 milliseconds. It allowed us to estimate each subject's true motor error distribution. Further, it provided subjects an opportunity to observe their own motor error.

Subjects started each trial by holding down the space bar of the keyboard with their index finger to trigger the presentation of the target. They were required to complete the reaching movement within the time limit. The movement time included the time from when the subject released the space bar to the time the finger touched the screen. If subjects completed the movement within the time limit, they would be informed whether it had been a hit or miss and a white dot would indicate the position of their end point. Otherwise, they would see a warning message.

We motivated subjects with performance-based bonuses and penalties. Subjects knew that at the end of the training phase that eight trials would be randomly drawn from the experimental trials they had just performed. For each of these bonus trials, they would win $1 for hit, zero for miss, or lose $1 for time-out.

There were 300 training trials. To reduce fatigue, subjects were encouraged to take a break whenever they were tired and were required to take a 2-minute break after every 100 trials. There were 50 additional warm-up trials for subjects to familiarize themselves with the touch screen and the time limit.

Probability choice. In the probability choice task, subjects chose which of two targets would be easier to hit if they attempted to hit it as they had hit targets in the training task. Subjects were instructed to imagine they would try to hit the targets from the same starting position and under the same time limit as they had just done in the training task. The probability choice task was conducted immediately after the training task while subjects' experience of the training task was still fresh.

The time course of the task is shown in Figure 2A. A trial began with a fixation cross. Two shapes, a circle and a rectangle, were displayed sequentially in a random order. Each display lasted for 0.5 sec and was separated by a 0.5 sec blank screen. Subjects were prompted to make the choice “which is easier to hit? 1^st^ or 2^nd^?” Responses were made by key press.

There were 10 different rectangles (Figure 2C). The width-to-height ratio of the rectangle was either 4∶1 (horizontal rectangle) or 1∶4 (vertical rectangle). The rectangle had five possible sizes, tuned to each subject's motor variance. We define the *mean standard deviation* of motor errors as 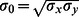, where 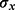 and 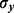 are respectively the standard deviations of the positions of the end points (time-out trials excluded) in the horizontal and vertical directions. The five possible short-side lengths of the rectangle formed a geometric sequence: 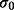, 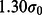, 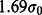, 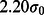, 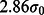.

The radius of the circle was adjusted by an adaptive staircase procedure. For each of the 10 rectangles, there were one 1-up/2-down and one 2-up/1-down staircases for its paired circle. The radius of the circle was increased or decreased multiplicatively. The step sizes were 0.115, 0.075, 0.05, 0.04 in log units, respectively for the first, second, third and the remaining reversals. A staircase terminated after 50 trials. All 20 staircases were interleaved with each other.

The 20 staircases ×50 = 1000 trials were run in blocks of 100 trials, preceded by 20 warm-up trials. Trials were self-initiated by a key press. Subjects were encouraged to take a break whenever tired. As an incentive, subjects were instructed that, at the end of the experiment, they would attempt to hit eight of the targets they preferred. They were rewarded for hits on these trials just as in the training task. The eight targets were randomly selected from the targets that they had judged to be easier to hit.

Area choice. The area choice task was a control to the probability choice task, where the same stimuli were used but now the task was to choose the alternative that was larger in area.

Subjects were rewarded for correct responses. Subjects were instructed that eight trials would be selected at random after they completed the 1000 trials. For each of the trials, subjects would win $1 if their choice had been correct.

### Experiment 2

#### Subjects

Twelve new naïve subjects, six female and six male, 11 right-handed and one left-handed, participated.

#### Apparatus and stimuli

Same as Experiment 1, except that no chinrest was used.

#### Procedure and design

Subjects completed three tasks in one session in the following order: *training*, *probability choice*, and *probability choice with feedback*. The first two tasks were the same as those of Experiment 1. The task of probability choice with feedback was similar to a combination of the tasks of probability choice and training. On each trial, subjects first chose between two sequentially displayed targets the one that was easier to hit. After that, they initiated a pointing trial by placing their index finger on the space bar and would try to hit the target they chose within the time limit. Feedback of the endpoint was given as in the training task. The bonus rule was the same as that of the training task.

In the two tasks of probability choice, same as Experiment 1, rectangles' sizes were tuned to each subject's motor variance and the radius of the circle was adjusted by adaptive staircase procedures. The design differed from Experiment 1 only in the sizes of rectangles and settings of staircase procedures. There were 8 (2 orientations by 4 sizes) different rectangles, whose short-side lengths were: 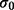, 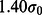, 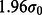, 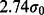. For each of the 8 rectangles, there was one 1-up/1-down staircase that terminated after 60 trials. (The motivation for us to adopt the one 1-up/1-down staircase for each rectangle rather than the one 1-up/2-down and one 2-up/1-down staircases in Experiment 1 was to use fewer trials to estimate each equivalent radius. This change would not introduce any known biases into the estimation of equivalent radius, given the data fitting procedures described below.) The step sizes were 0.15, 0.1, 0.08, 0.06 in log units, respectively for the first, second, third and the remaining reversals. Interleaved, the 8 staircases ×60 = 480 trials were run in blocks of 60 trials, preceded by 8 warm-up trials.

### Data fitting

#### Equivalent radius

For a specific rectangle in a choice task, we defined the *equivalent radius* (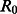) as the radius of the circle such that the subject was indifferent between the rectangle and the circle in her choice. The method of eliciting the equivalent radius was as follows.

For each subject and each specific rectangle, we assumed that the probability of choosing the circle was a Quick-Weibull psychometric function [26], [27] of the radius of the circle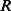:
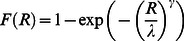
(5)where 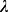 is a position parameter and 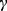 is a steepness parameter. Assuming that different rectangles were associated with different 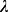 but a common 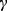, we estimated 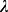 and 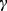 for each rectangle by fitting the responses of the staircase trials to Eq. 5 using maximum likelihood estimates.

By the definition of equivalent radius, 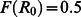. Substituting into Eq. 5 and solving it, we have
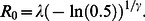
(6)Our estimates of equivalent radius were based on our estimates of 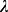 and 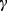 for each subject substituted into Eq. 6.

We verified that staircases converged for subjects of the area-matching type as well as for subjects of the Gaussian type (Figure S2). The steepness parameter 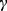 in Eq. 5 can serve as an indicator of the subject's choice consistency. The larger the 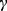, the more likely the subject would make the same choice between a specific rectangle and a specific circle. We compared the choice consistency of subjects of the Gaussian type to that of the area-matching type. According to two-sample, two-tailed Student's *t*-tests, the 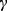 elicited from the probability choice task was indistinguishable between the two groups in both Experiment 1, *t*(16) = 0.81, *p* = .43, and Experiment 2, whether in choices with no feedback, *t*(10) = 0.87, *p* = .40, or with feedback, *t*(10) = −0.40, *p* = .70.

#### Gaussian model

For each subject, we used their equivalent radii to estimate the variance parameter 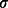 and the anisotropy parameter 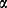 in the bivariate Gaussian probability density function (Eq. 4). By definition, each rectangle (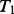) and its equivalent circle (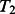) were perceived to be equivalent in probability of hit:
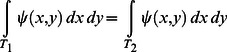
(7)Substituting Eq. 4 into Eq. 7, we have an equation of 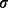 and 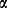:
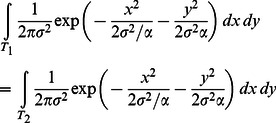
(8)That is, for any specific rectangle, we could compute its equivalent radius as a function of 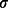 and 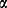 based on Eq. 8. We assumed that the logarithms of the measured equivalent radii deviated from the predicted equivalent radii by random additive Gaussian noise 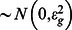. We used maximum likelihood estimates to fit the measured equivalent radii to obtain 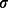, 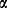, 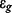.

#### Area-matching model and model comparison

We tested area-matching as an alternative model for subjects' choices. That is, the subject would choose the target of a larger area, as they should do in the area choice task. In particular, we assumed that the logarithms of the measured equivalent radii deviated from the logarithms of the true area equivalent radii only by a random noise 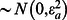. We fitted 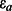 using maximum likelihood estimates.

The area-matching model is a special case of the Gaussian model (Eq. 4): when 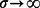 and 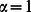, the Gaussian model would result in the same choices as the area-matching model does. We used nested hypothesis tests [12] to test the Gaussian model against the area-matching model. Denote the log likelihoods of the Gaussian model and the area-matching model as 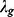 and 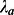. We computed a test statistic 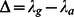. If the model with fewer parameters – the area-matching model – is the correct model then this test statistic is asymptotically distributed as a 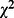 random variable with degrees of freedom equal to the difference in number of parameters in the two models under comparison. Accordingly we compared 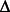 to the 95^th^ percentile of a 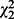 distribution.

#### Confidence intervals

We computed the 95% confidence intervals of 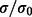, 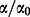, 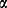, 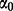 using a bootstrap method [28]. For each subject, we ran a virtual experiment for 1000 times and estimated the above measures on each run. In the training task, endpoint positions were resampled from the non-time-out trials. In the probability choice task, the responses of each staircase trial was generated by parametric resampling [29] from the psychometric functions (Eq. 5) that was fitted with the real data.

## Supporting Information

Figure S1
**Illustration of the difficulty in estimating** 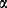 **as** 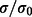 **increases.** For the specific rectangle conditions (proportional to 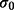) in Experiment 1, the equivalent radii 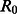 are computed for a virtual observer who assumes an error distribution in the form of Eq. 4 with parameters 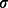 and 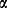. The predicted 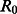 for 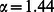 and for 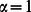 are plotted against each other to show how the virtual observer's 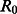 would differ for different 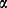 when the 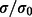 is the same. The identity line corresponds to no difference at all. Each panel is plotted for a different 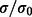. Note that as the 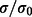 increases, the effect of varying 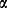 diminishes. In the real experiment, at the existence of response noise, a smaller difference implies less discriminability. That is, 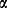 could not be precisely determined when 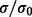 is large enough.(TIFF)

Figure S2 **Staircase convergence in the probability choice task of Experiment 1.** The radius of the circle was plotted as a function of the trial No. of each staircase for typical subjects of the Gaussian type (left) and the area-matching type (right). Top panels are for horizontal rectangles; bottom panels for vertical rectangles. Blue circles and red X's denote 1-up/2-down and 2-up/1-down staircases. Visually scrutinized, staircases of both subjects were well converged (see the Methods for a formal comparison of staircase convergence between the two types of subjects).
(TIFF)
