# Supplementary figures and images for: Testing Whether Humans Have an Accurate Model of Their Own Motor Uncertainty in a Speeded Reaching Task
Source: PLoS Comput Biol. 2013 May 23;9(5):e1003080. doi: 10.1371/journal.pcbi.1003080 (PMC3662689; doi:10.1371/journal.pcbi.1003080)

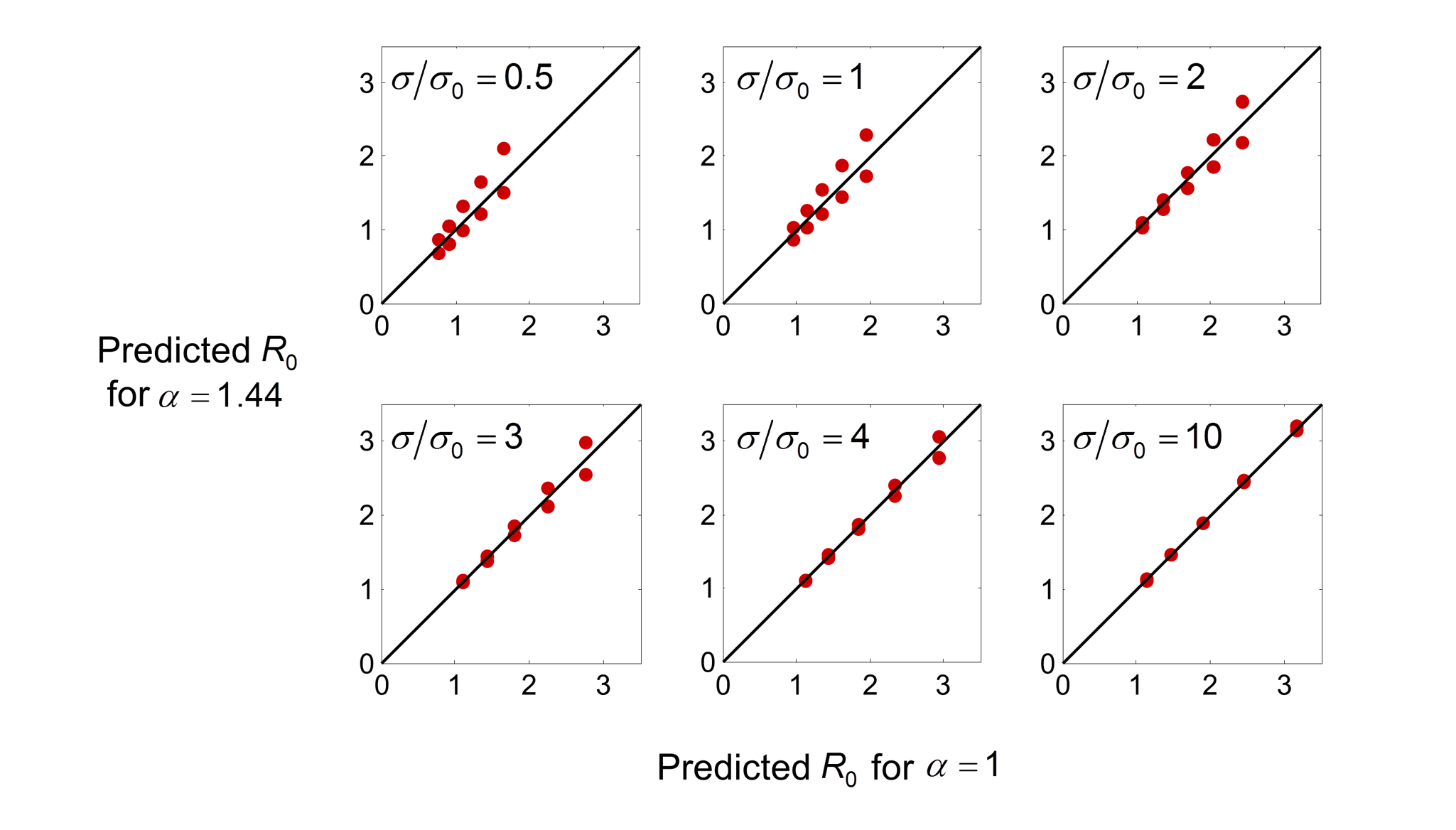

Supplement: Figure S1 — Illustration of the difficulty in estimating as increases. For the specific rectangle conditions (proportional to ) in Experiment 1, the equivalent radii are computed for a virtual observer who assumes an error distribution in the form of Eq. 4 with parameters and . The predicted for and for are plotted against each other to show how the virtual observer's would differ for different when the is the same. The identity line corresponds to no difference at all. Each panel is plotted for a different . Note that as the increases, the effect of varying diminishes. In the real experiment, at the existence of response noise, a smaller difference implies less discriminability. That is, could not be precisely determined when is large enough. (TIFF) [file pcbi.1003080.s001.tiff]

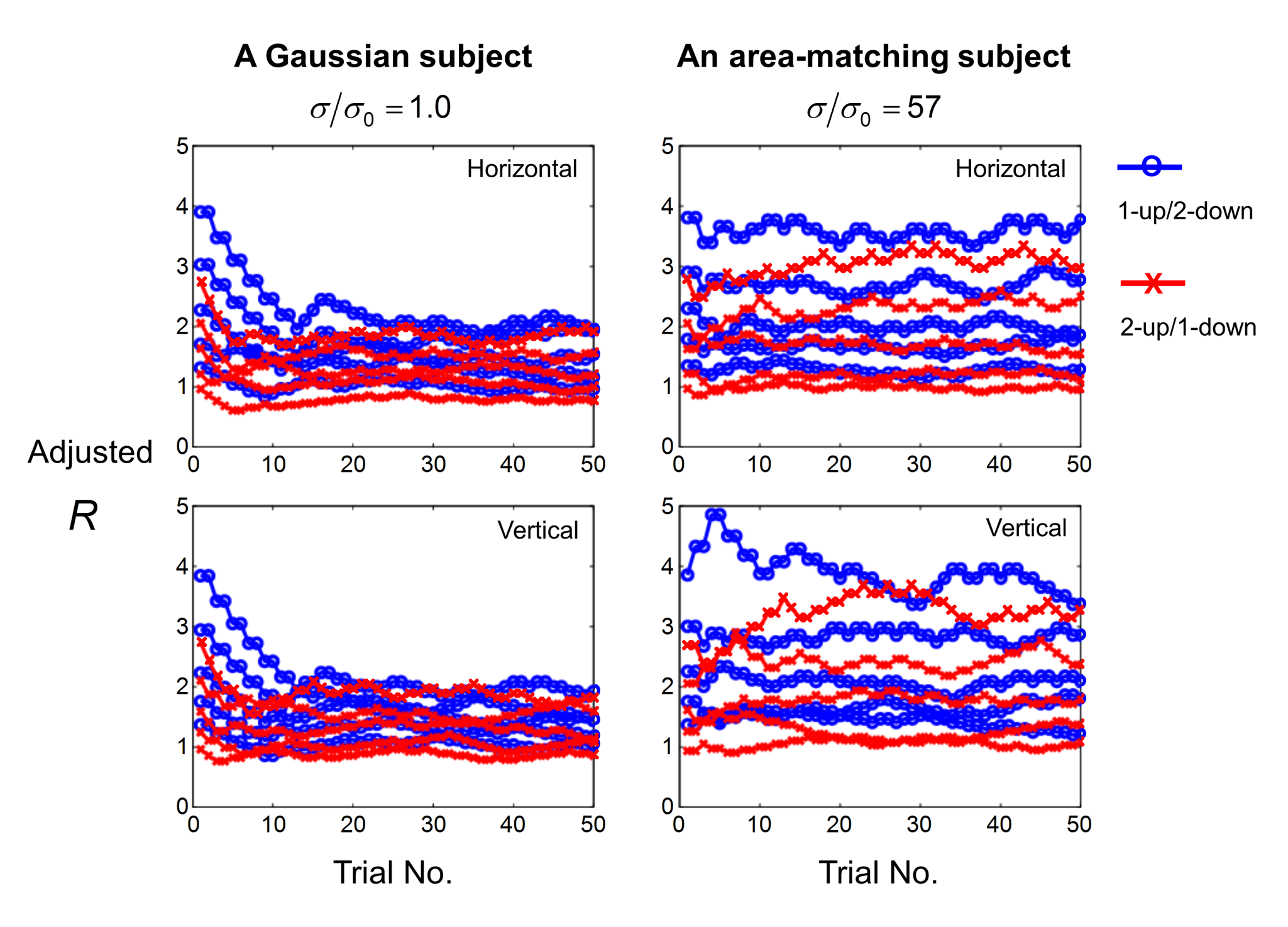

Supplement: Figure S2 — Staircase convergence in the probability choice task of Experiment 1. The radius of the circle was plotted as a function of the trial No. of each staircase for typical subjects of the Gaussian type (left) and the area-matching type (right). Top panels are for horizontal rectangles; bottom panels for vertical rectangles. Blue circles and red X's denote 1-up/2-down and 2-up/1-down staircases. Visually scrutinized, staircases of both subjects were well converged (see the Methods for a formal comparison of staircase convergence between the two types of subjects). (TIFF) [file pcbi.1003080.s002.tiff]
